# Supplementary material for: XPO5 promotes primary miRNA processing independently of RanGTP
Source: Nat Commun. 2020 Apr 15;11:1845. doi: 10.1038/s41467-020-15598-x (PMC7160132; doi:10.1038/s41467-020-15598-x)
Supplement: Supplementary file 1 — Supplementary Information [file 41467_2020_15598_MOESM1_ESM.pdf]

XPO5 promotes primary miRNA processing independently of RanGTP

Wang *et al*.

Supplementary Figure 1

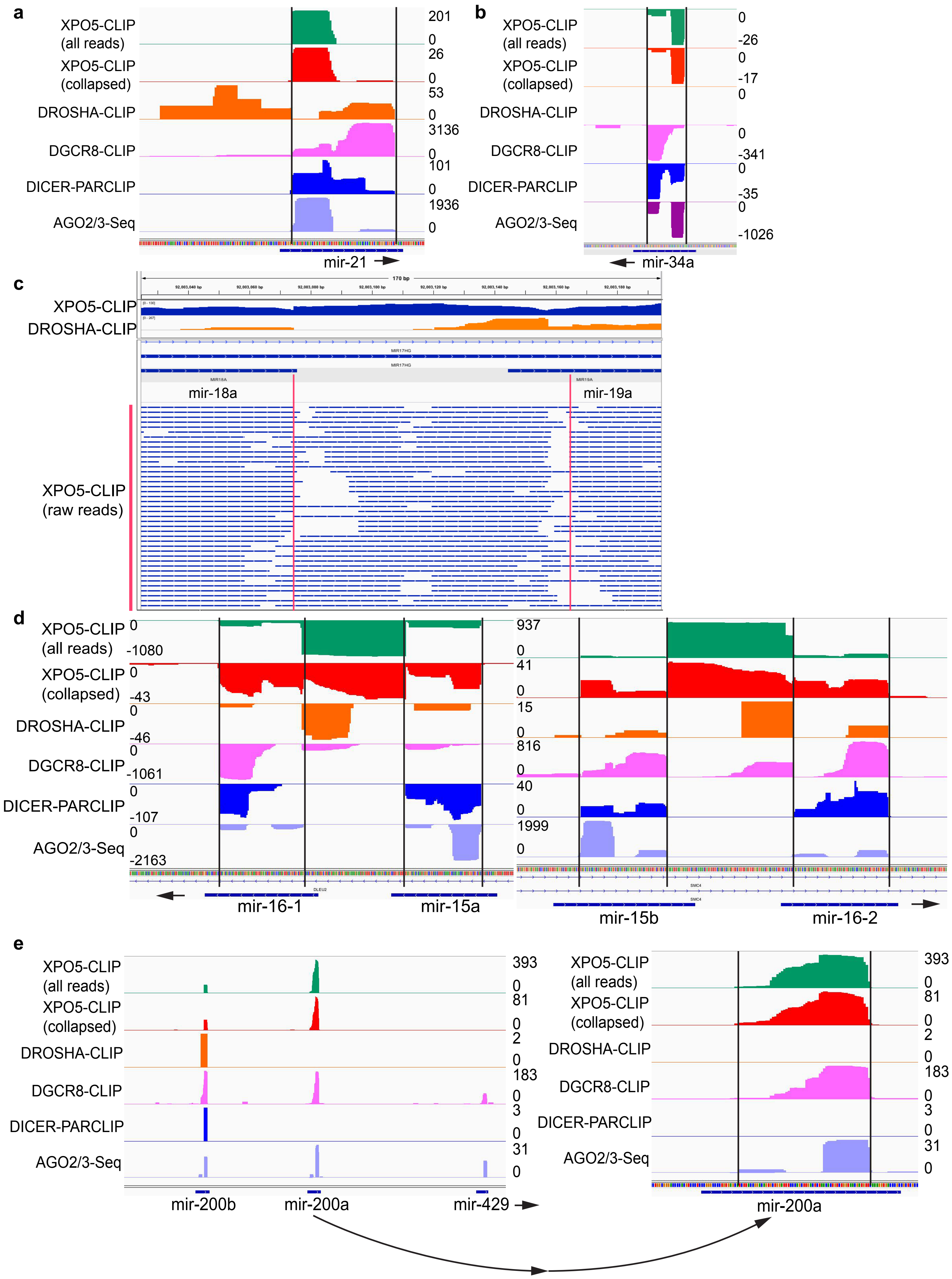

Supplementary Figure 1. XPO5 associates with primary transcripts of closely clustered miRNAs.
(a-b) IGV tracks of XPO5-CLIP, DROSHA-CLIP, DGCR8-CLIP, DICER-PARCLIP and AGO2/3-Seq for *mir-21* (a) and *mir-34a* (b). XPO5 binding to these monocistronic miRNAs is restricted within the pre-miRNA hairpin.
(c) Individual reads of XPO5 HITS-CLIP data show that XPO5 recognizes uncleaved *pri-mir-17~92* fragments between *mir-18a* and *mir-19a*. Red lines indicate the boundary of pre-miRNA hairpin. Note that many reads are across the DROSHA cleaving sites. (d-e) IGV tracks of XPO5-CLIP, DROSHA-CLIP, DGCR8-CLIP, DICER-PARCLIP and AGO2/3-Seq for *mir-15a~16-1* and *mir-15b~16-2* clusters (d), and *mir-200b* cluster (e). Widespread inter-pre-miRNA reads are only detected in *mir-15a~16-1* and *mir-15b~16-2* clusters but not in *mir-200b* cluster. Blue bars at the bottom and black lines together indicate the location of pre-miRNAs. Data range is shown on the left or right of each track. Negative value indicates the reads mapping to the minus strand.

Supplementary Figure 2

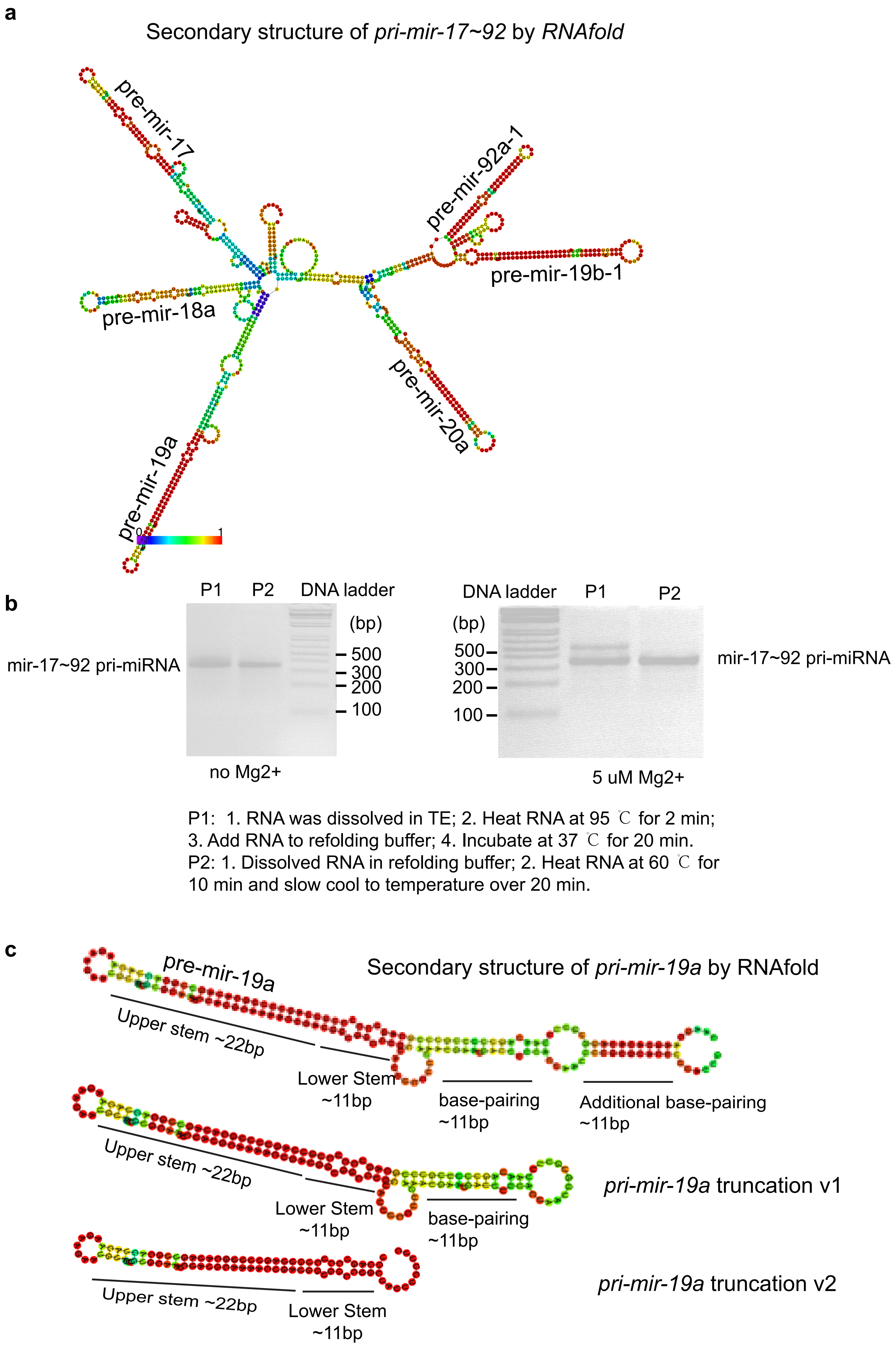

Supplementary Figure 2. Predicted secondary structure and folding of *pri-mir-17~92* and *pri-mir-19a*. (a) Secondary structure of *pri-mir-17~92* is predicted by RNAfold. Heat map indicates the probability of secondary structures (Purple: 0 - low; Red: 1 - high). (b) *Pri-mir17~92* is in vitro transcribed and folded under P1 and P2 conditions in refolding buffer with or without Mg2+. Note that a DNA ladder is used, which does not reflect the correct size of RNA. (c) Secondary structure of *pri-mir-19a* and its truncation versions are predicted by RNAfold. Heat map indicates the probability of secondary structures (Purple: 0 - low; Red: 1 - high). Original data for b are provided in the Source Data file.

Supplementary Figure 3

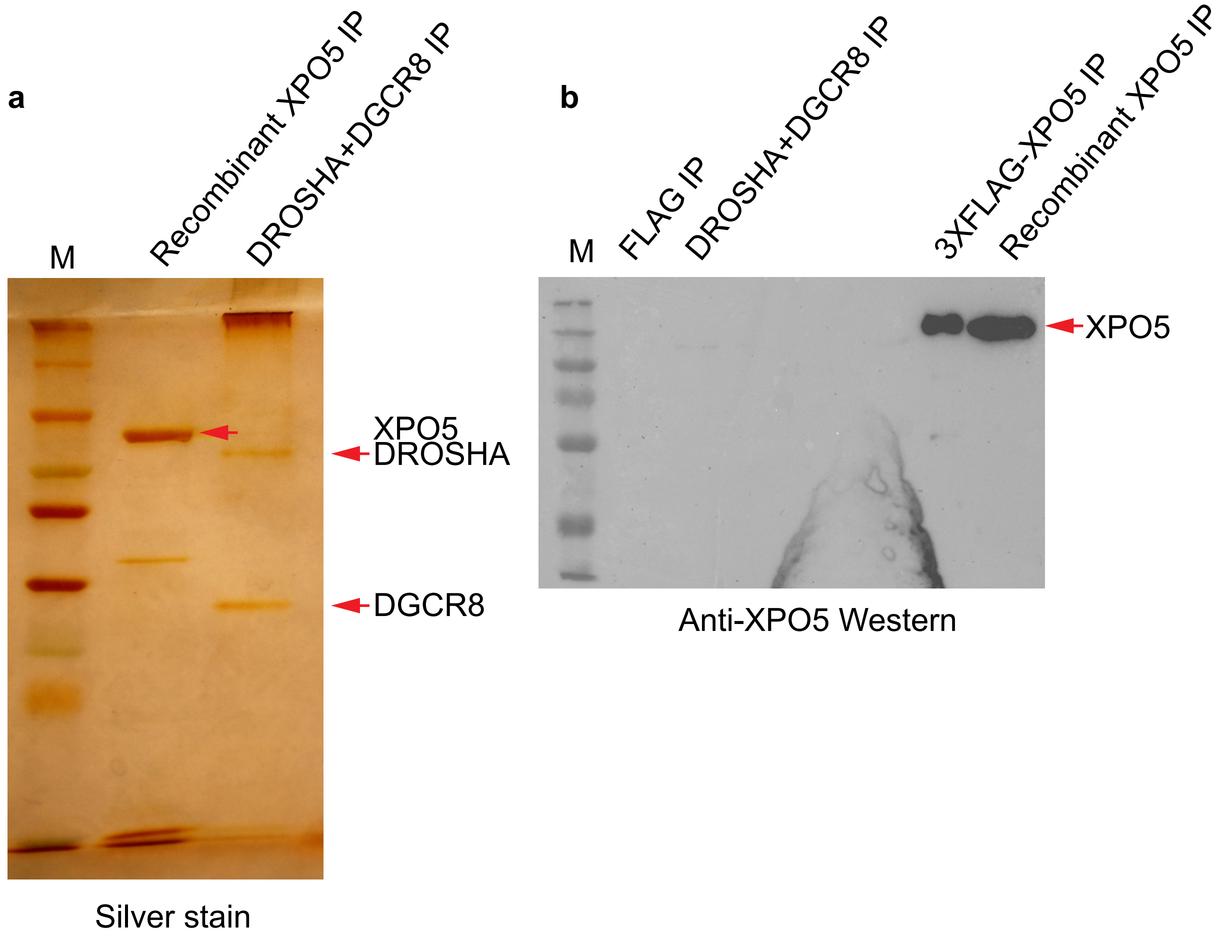

Supplementary Figure 3. XPO5 does not associate with the microprocessor.
(a) No XPO5 band is detected by silver staining for the 3XFLAG tagged microprocessor (DROSHA and DGCR8) immunoprecipitation sample from HEK293T cells. (b) Lack of XPO5 associating with the microprocessor is confirmed by XPO5 Western blotting for 3XFLAG tagged microprocessor immunoprecipitation sample. Both 3XFLAG-XPO5 and recombinant XPO5 are recognized by XPO5 antibody.

Supplementary Figure 4

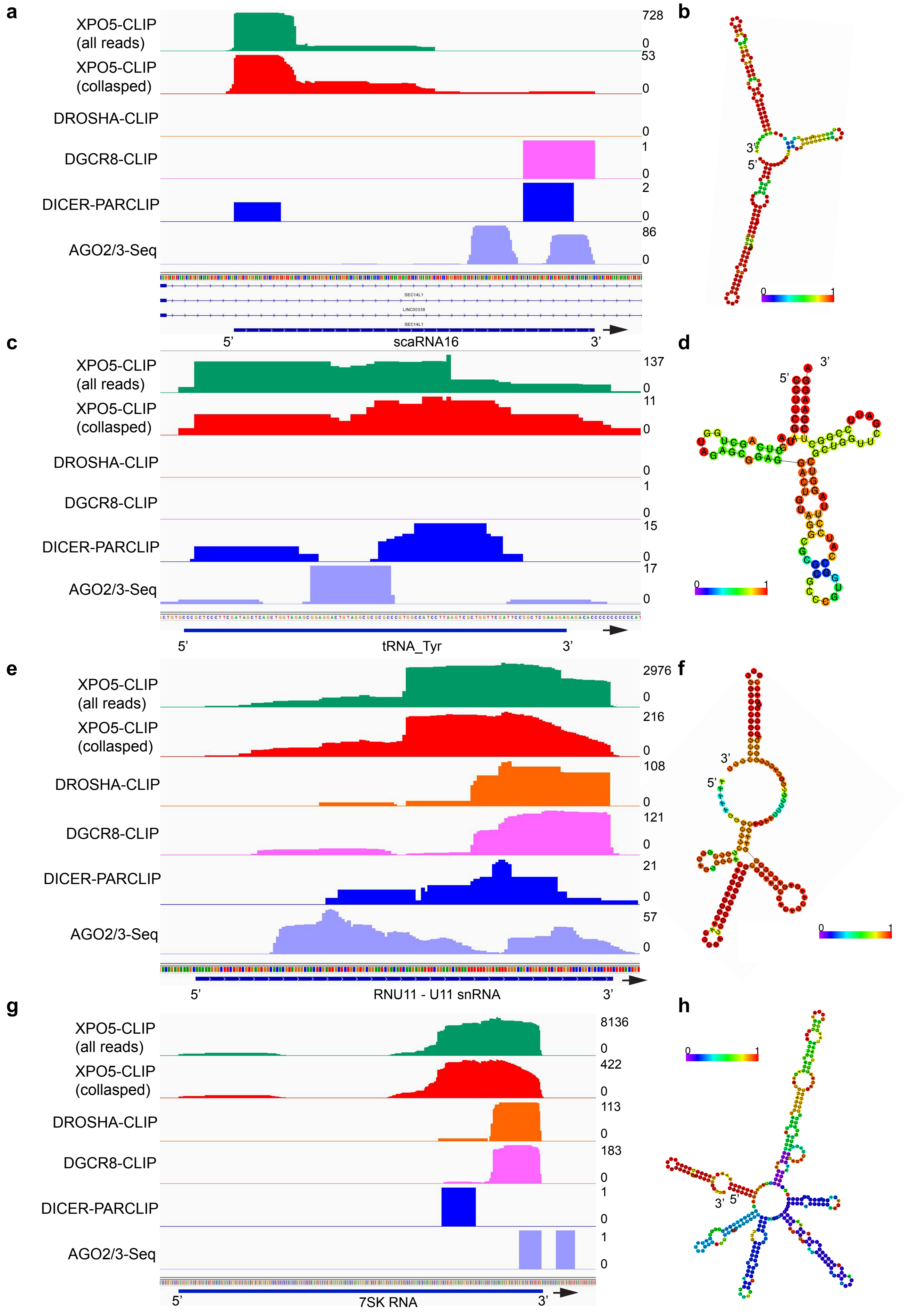

Supplementary Figure 4. XPO5 associates with diverse cellular RNAs containing double-stranded regions.
(a, c, e, g) IGV tracks of XPO5-CLIP, DROSHA-CLIP, DGCR8-CLIP, DICER-PARCLIP and AGO2/3-Seq show the association of each component with scaRNA16 (a), tRNA-Tyr (c), U11 snRNA (e) and 7SK RNA (g). Blue bars at the bottom indicate the coding region of each RNA. Arrows indicate the direction of transcription. Data range is shown on the right of each track. (b, d, f and h) The predicted secondary structure of each RNA by *RNAfold*. Heat map indicates the probability of secondary structures (Purple: 0 - low; Red: 1 - high).

Supplementary Figure 5

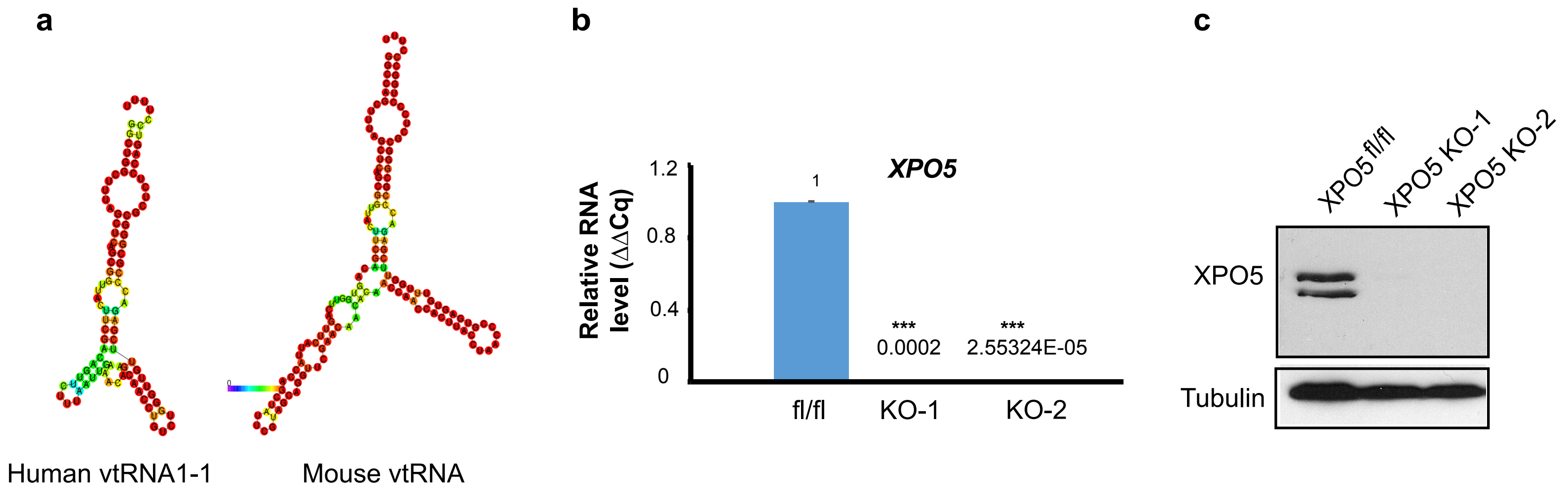

Supplementary Figure 5. Predicted secondary structure of human vtRNA1-1 and mouse vault RNA and confirmation of XPO5 knock-out MEF cells.
(a) Secondary structures of human vtRNA1-1 and mouse vault RNA are predicted by *RNAfold*. Heat map indicates the probability of secondary structures (Purple: 0 - low; Red: 1 - high). (b-c) Depletion of XPO5 RNA and protein in *XPO5* KO MEF cells are confirmed by qPCR and Western blot. Data shown are mean s.d. from 3 independent experiments. *** P <0.001 by Student's t -test. Original data for c are provided in the Source Data file.

Supplementary Table 1 Antibodies and Primers

**Antibodies**	SOURCE	IDENTIFIER
Beta-4 antibody	BD Bioscience	Cat#553745
Anti-Ki67 antibody	Abcam	Cat#ab15580
Polyclonal Antibody against Keratin 5, K5 (AF 138)	Covance	Cat#PRB-160P
Purified anti-Keratin 1 Antibody	Covance	Cat#PRB-165P
LEF1 (C12A5) Rabbit mAb	Cell signaling	Cat#2230
Anti-Exportin-5 antibody [EPR8453]	Abcam	Cat#ab131281
ANTI-FLAG® M2 Affinity Gel	Sigma	Cat#A2220
Anti-Mouse Ago2, Monoclonal Antibody	Wako Chemicals GmbH	Cat#018-22021
β-Tubulin Antibody	Cell signaling	Cat#2146
**Primers**		
Names	sequences	
Human *XPO5* cloning forward primer	CCGCTCGAGGA ATGGCGATGGATC<br>AAGTAAACGC	
Human *XPO5* cloning reverse primer	GGCTCTAGATC AGGGTTCAAAGAT GGTGGCC	
miR17~92a pri-miRNA transcription forward primer	GCGGTACCTAATACGACTCACTATAGGGTCAG<br>A ATAATGTCA AAGTGCTTACAG	
miR17~92a pri-miRNA transcription reverse primer	GCAAGCTTCCAAACTCAACAGGCCGGGAC	
Pre-miR30a in vitro transcription forward primer	TAATACGACTCACTATAGGTAA ACATCCTCG<br>ACTGGAAGCTGT	
Pre-miR30a in vitro transcription reverse primer	GCTGCA AACATCCGACTGAAAGC	
Pre-miR19a in vitro transcription forward primer	TAATACGACTCACTATAGG<br>AGTTTTGCATAGTTGCACTACAAG	
Pre-miR19a in vitro transcription reverse primer	TCAGTTTTGCATAGATTTGCACAAC	
Pri-miR19a in vitro transcription forward primer	TAATACGACTCACTATAGGCAAGCAAGTATA<br>TAGGTGTTTTAATAG	
Pri-miR19a in vitro transcription reverse primer	CAATAAAAGTACACAAAATTAGTAAAAATCA	
Pri-mir-19a_truncation v1 in vitro transcription forward primer	TAATACGACTCACTATAGG TGT TTT AAT AGT<br>TTT TGT TTG CAG TCC TC	
Pri-mir-19a_truncation v1 in vitro transcription reverse primer	ATT AGT AAA AAT CAT TCA TTT GAA GGA AAT<br>AGC	
Pri-mir-19a_truncation v2 in vitro transcription forward primer	TAATACGACTCACTATAGG T GCA GTC CTC<br>TGT TAG TTT TGC ATA GTT G	
Pri-mir-19a_truncation v2 in vitro transcription reverse primer	GAA GGA AAT AGC AGG CCA CCA TC	
Pri-miR15b-16-2 in vitro transcription forward primer	TAATACGACTCACTATAGGTTGAGGCCTTAA<br>AGTACTGTAGC	
Pri-miR15b-16-2 in vitro transcription reverse primer	TCC CTG TCA CAC TAA AGC AGC	
Pri-miR15b in vitro transcription reverse primer	CAT AGT TTT GAA TGA ATT TCC TTA AAT	
VtRNA1-1 in vitro transcription forward primer	TAATACGACTCACTATAGGGCTGGCTTTAG<br>CTCAGCGG	
VtRNA1-1 in vitro transcription reverse primer	AAAAGGACTGGAGAGCGCCCG	
Mouse vtRNA probe for northern blot	GTA ACC GCT GAG CTA AAG CTG GCC	
Mouse 5S RNA probe for northern blot	TCAGACGAGATCGGGCGCGTTCAGGGTGGT	
**qPCR primers**		
*XPO5*_q_for primer	TGATCCTGTTTGGAGATGTCG	
*XPO5*_q_rev primer	CACATAGCAGATTTCCCAGTG	
*Oct4*_q_for primer	AGTGGAAAGCAACTCAGAGG	
*Oct4*_q_rev primer	AACTGTTCTAGCTCCTTCTGC	

*T*_q_for primer	CCATTGCTCACAGACCAGAG	
*T*_q_rev primer	GTCTAGCCTCGGAGTGCCT	
*Nanog*_q_for primer	TCCAAGTTGGGTTGGTCCAAGTCT	
*Nanog*_q_rev primer	AACCAAAGGATGAAGTGCAAGCGG	
*Tet*_q_for primer	TTGCTCTTCTTCCCCATGAC	
*Tet*_q_rev primer	CGAAAGAACAGCCACCAGAT	
*Krt5*_q_for_primer	GAGTTT GTG ATG TTG AAG AAG GAT GTG G	
*Krt5*_q_rev_primer	CCA TCA GGG CAT CGA CCC TG	
*Krt1*_q_for_primer	TCA GAT TCA AAA AGT GAA GTC TCA GGA	
*Krt1*_q_rev_primer	CGG GTT GTG GTG TCT ACC TGC T	
*Lor*_q_for_primer	AAG CAG CGG CGG CTC TAG	
*Lor*_q_rev_primer	CTA GAA CCG CCT CCG TAG CT	
*Itgb4*_q_for_primer	TTGGTCGCCGTCTGGTAAAC	
*Itgb4*_q_rev_primer	GTGCCGGATGACAGGGATG	
Recombinant DNA		
pCK-Drosha-FLAG plasmid	(Lee et al., 2003)	N/A
pCK-FLAG-Dgcr8 plasmid	(Lee et al., 2003)	N/A
pCDNA3-FLAG-XPO5 plasmid	This paper	N/A
pCDNA3-FLAG-RanQ69L plasmid	This paper	N/A
pQE60-*XPO5* plasmid	Addgene	Cat#12553
pQE32-*RanQ69L* plasmid	Gift from Ian G. Macara	N/A
pJMC101-T7-pri-miR17~92	This paper	N/A
**Software and Algorithms**		
The mfold Web Server	Michael Zuker & Nick Markham	N/A
Cutadapt	(Martin, 2011)	https://cutadapt.readthedocs.io/en/stable/
BedTools (v2.26.0)	(Quinlan and Hall, 2010)	http://bedtools.readthedocs.io/en/latest/
Bowtie2 (v.2.1.0)	John Hopkins University	http://bowtie-bio.sourceforge.net/bowtie2/index.shtml
Htseq-count (v.0.6.0)	(Anders et al., 2015)	https://htseq.readthedocs.io/en/release_0.9.1/
Novoalign	Novocraft	http://www.novocraft.com/products/novoalign/
