# Supplementary material for: XPO5 promotes primary miRNA processing independently of RanGTP
Source: Nat Commun. 2020 Apr 15;11:1845. doi: 10.1038/s41467-020-15598-x (PMC7160132; doi:10.1038/s41467-020-15598-x)
Supplement: Supplementary file 3 — Reporting Summary [file 41467_2020_15598_MOESM3_ESM.pdf]

nature research

Corresponding author(s): Rui Yi

Last updated by author(s): Feb 25, 2020

Reporting Summary

Nature Research wishes to improve the reproducibility of the work that we publish. This form provides structure for consistency and transparency in reporting. For further information on Nature Research policies, see Authors & Referees and the Editorial Policy Checklist.

Statistics

For all statistical analyses, confirm that the following items are present in the figure legend, table legend, main text, or Methods section.

n/a	Confirmed	
☐	☒	The exact sample size (*n*) for each experimental group/condition, given as a discrete number and unit of measurement
☒	☐	A statement on whether measurements were taken from distinct samples or whether the same sample was measured repeatedly
☐	☒	The statistical test(s) used AND whether they are one- or two-sided<br>*Only common tests should be described solely by name; describe more complex techniques in the Methods section.*
☒	☐	A description of all covariates tested
☒	☐	A description of any assumptions or corrections, such as tests of normality and adjustment for multiple comparisons
☒	☐	A full description of the statistical parameters including central tendency (e.g. means) or other basic estimates (e.g. regression coefficient) AND variation (e.g. standard deviation) or associated estimates of uncertainty (e.g. confidence intervals)
☐	☒	For null hypothesis testing, the test statistic (e.g. *F*, *t*, *r*) with confidence intervals, effect sizes, degrees of freedom and *P* value noted<br>*Give P values as exact values whenever suitable.*
☒	☐	For Bayesian analysis, information on the choice of priors and Markov chain Monte Carlo settings
☒	☐	For hierarchical and complex designs, identification of the appropriate level for tests and full reporting of outcomes
☒	☐	Estimates of effect sizes (e.g. Cohen's *d*, Pearson's *r*), indicating how they were calculated

Our web collection on statistics for biologists contains articles on many of the points above.

Software and code

Policy information about availability of computer code

Data collection	Details are described in Methods section.
Data analysis	XPO5 HITS-CLIP and Small RNA-seq analysis are described in Methods section. In vitro processing assay and Electrophoretic mobility shift assay were analyzed by ImageJ. Binding affinity Kd of XPO5 with its substrates were calculated by Prism 8.0.2.

For manuscripts utilizing custom algorithms or software that are central to the research but not yet described in published literature, software must be made available to editors/reviewers. We strongly encourage code deposition in a community repository (e.g. GitHub). See the Nature Research guidelines for submitting code & software for further information.

Data

Policy information about availability of data

All manuscripts must include a data availability statement. This statement should provide the following information, where applicable:

- Accession codes, unique identifiers, or web links for publicly available datasets
- A list of figures that have associated raw data
- A description of any restrictions on data availability

Web links are listed in the Methods section and Supplementary Table 1. XPO5 HITS-CLIP data and small RNA-seq data are deposited in the Gene Expression Omnibus (GEO) data repository with accession number GSE111964. The source data underlying Figs 1a, 2a–c, 2e, 2h, 2j, 3b, 3d, 3f-g, 4a-c, 5c, 5h, 7d, 7e, 7g and Supplementary Figs 2b and 3c are provided as a Source Data file.

Field-specific reporting

Please select the one below that is the best fit for your research. If you are not sure, read the appropriate sections before making your selection.

☒ Life sciences ☐ Behavioural & social sciences ☐ Ecological, evolutionary & environmental sciences

For a reference copy of the document with all sections, see nature.com/documents/nr-reporting-summary-flat.pdf

Life sciences study design

All studies must disclose on these points even when the disclosure is negative.

Sample size	For animal study, 3 animals are set as the minimal sample size.
Data exclusions	No data exclusions in the study
Replication	Experiments were repeated with similar results at least two times with independent biological replicates. Individual repeats and sample sizes as well as significance levels are indicated in the Figure legends; statistical significance was determined as described in Figure legends.
Randomization	Sample randomization is not applicable to studies presented here.
Blinding	Not applicable

Reporting for specific materials, systems and methods

We require information from authors about some types of materials, experimental systems and methods used in many studies. Here, indicate whether each material, system or method listed is relevant to your study. If you are not sure if a list item applies to your research, read the appropriate section before selecting a response.

Materials & experimental systems

n/a	Involved in the study	
☐	☒	Antibodies
☐	☒	Eukaryotic cell lines
☒	☐	Palaeontology
☐	☒	Animals and other organisms
☒	☐	Human research participants
☒	☐	Clinical data

Methods

n/a	Involved in the study	
☒	☐	ChIP-seq
☒	☐	Flow cytometry
☒	☐	MRI-based neuroimaging

Antibodies

Antibodies used	For details please see details in Supplementary Table 1
Validation	For details please see details in Supplementary Table 1

Eukaryotic cell lines

Policy information about cell lines

Cell line source(s)	HEK293T cells were obtained from ATCC. XPO5 fl/fl MEF cell lines are extracted from E14 XPO5fl/fl embryos and maintained in the lab.
Authentication	No specific cell line authentication was performed.
Mycoplasma contamination	HEK293T cells and MEF cells were not tested for Mycoplasma contamination.
Commonly misidentified lines (See ICLAC register)	No misidentified lines used.

Animals and other organisms

Policy information about studies involving animals; ARRIVE guidelines recommended for reporting animal research

Laboratory animals	XPO5KI mice, XPO5 fl/fl mice, K14-Cre/XPO5 fl/fl mice

Wild animals	No wild animals are used in the study.
Field-collected samples	No field-collected samples are used in the study.
Ethics oversight	Ethic Committees at the authors' institutes, for details please see the Methods section.

Note that full information on the approval of the study protocol must also be provided in the manuscript.
