# Supplementary material for: XPO5 promotes primary miRNA processing independently of RanGTP
Source: Nat Commun. 2020 Apr 15;11:1845. doi: 10.1038/s41467-020-15598-x (PMC7160132; doi:10.1038/s41467-020-15598-x)
Supplement: Supplementary file 4 — Description of Additional Supplementary Files [file 41467_2020_15598_MOESM4_ESM.pdf]

Description of Additional Supplementary Files

File Name: Supplementary Data 1
Description: miRNA sequencing of HEK293T cells

File Name: Supplementary Data 2
Description: HITS-CLIP identifies XPO5 associated cellular RNAs in HEK293T cells

File Name: Supplementary Data 3
Description: smRNA-seq of XPO5 control and cKO epidermal cells
